# CMS: A Web-Based System for Visualization and Analysis of Genome-Wide Methylation Data of Human Cancers

**DOI:** 10.1371/journal.pone.0060980

**Published:** 2013-04-22

**Authors:** Fei Gu, Mark S. Doderer, Yi-Wen Huang, Juan C. Roa, Paul J. Goodfellow, E. Lynette Kizer, Tim H. M. Huang, Yidong Chen

**Affiliations:** 1 Department of Molecular Medicine/Institute of Biotechnology, University of Texas Health Science Center at San Antonio, San Antonio, Texas, United States of America; 2 Greehey Children's Cancer Research Institute, University of Texas Health Science Center at San Antonio, San Antonio, Texas, United States of America; 3 Department of Obstetrics and Gynecology, Medical College of Wisconsin, Milwaukee, Wisconsin, United States of America; 4 Departamento de Pathología, Universidad de la Frontera, Temuco, Fono, Chile; 5 Department of Obstetrics and Gynecology, University of Texas Health Science Center at San Antonio, San Antonio, Texas, United States of America; 6 Department of Surgery, Washington University School of Medicine and Siteman Cancer Center, St. Louis, Missouri, United States of America; 7 Department of Epidemiology and Biostatistics, University of Texas Health Science Center at San Antonio, San Antonio, Texas, United States of America; 8 Cancer Therapy & Research Center, University of Texas Health Science Center at San Antonio, San Antonio, Texas, United States of America; National Taiwan University, Taiwan

## Abstract

**Background:**

DNA methylation of promoter CpG islands is associated with gene suppression, and its unique genome-wide profiles have been linked to tumor progression. Coupled with high-throughput sequencing technologies, it can now efficiently determine genome-wide methylation profiles in cancer cells. Also, experimental and computational technologies make it possible to find the functional relationship between cancer-specific methylation patterns and their clinicopathological parameters.

**Methodology/Principal Findings:**

Cancer methylome system (CMS) is a web-based database application designed for the visualization, comparison and statistical analysis of human cancer-specific DNA methylation. Methylation intensities were obtained from MBDCap-sequencing, pre-processed and stored in the database. 191 patient samples (169 tumor and 22 normal specimen) and 41 breast cancer cell-lines are deposited in the database, comprising about 6.6 billion uniquely mapped sequence reads. This provides comprehensive and genome-wide epigenetic portraits of human breast cancer and endometrial cancer to date. Two views are proposed for users to better understand methylation structure at the genomic level or systemic methylation alteration at the gene level. In addition, a variety of annotation tracks are provided to cover genomic information. CMS includes important analytic functions for interpretation of methylation data, such as the detection of differentially methylated regions, statistical calculation of global methylation intensities, multiple gene sets of biologically significant categories, interactivity with UCSC via custom-track data. We also present examples of discoveries utilizing the framework.

**Conclusions/Significance:**

CMS provides visualization and analytic functions for cancer methylome datasets. A comprehensive collection of datasets, a variety of embedded analytic functions and extensive applications with biological and translational significance make this system powerful and unique in cancer methylation research. CMS is freely accessible at: http://cbbiweb.uthscsa.edu/KMethylomes/.

## Introduction

DNA methylation of promoter CpG islands is associated with gene suppression in tumor samples comparing to normal counterpart, and its unique genome-wide profiles have been linked to tumor progression and can be used to predict patient survival [1]. Global hypomethylation was detected in breast and colon tumors comparing with corresponding normal tissues [2], [3]. More specifically, in breast cancer, it has been shown that gene body hypomethylation is associated with gene silencing, while hypermethylation of regions close to a transcription start site (TSS) tends to cause a similar effect [4]. In addition, interplay between DNA methylation and transcription factors (TFs) are important for regulating human cell phenotypes. With the advancement of sequencing technology, large-scale analysis of genome-wide methylation becomes feasible. Several experimental methods have been developed to capture methylated DNAs, including MeDIP [5], MBD [6], MethylC [7], and RRBS [8]. Coupled with high-throughput sequencing technologies, these methods can now efficiently determine genome-wide methylation profiles in cells. Moreover, various computational and statistical methods have been proposed for the analysis of differentially methylated regions (DMR). These experimental and computational technologies make it possible to find the functional relationship between cancer-specific methylation patterns and gene suppression, and their association with clinicopathological parameters, leading to the identification of candidate biomarkers for diagnosis and prognosis [9].

Here we describe a novel cancer methylome system which systematically collects, organizes, visualizes and analyzes a large set of DNA methylation data by sequencing from human endometrial and breast cancers. The datasets are obtained by using MBDCap-seq protocol, a technique used to capture methylated DNAs by using a methyl-CpG binding domain (MBD) protein column followed by next-generation sequencing [10]. The low cost and unbiased display of methylation profiles of both CpG and non-CpG island regions make it suitable for genome-wide methylation profile analysis. 191 patient samples (169 tumor and 21 normal specimen) and 41 breast cancer cell-lines were processed with the MBDCap-seq protocol, generating a total of about 6.6 billion uniquely mapped sequence reads. Datasets were pre-processed and stored in a MySQL database. CMS offers user-friendly tools for rapid identification of differentially methylated regions (DMRs) among different groups of samples (e.g., normal versus tumor), regardless of their gene proximity. Methylation intensities were generated for both genome-wide (resolution in 100 bp) and gene (for every RefSeq annotated gene) levels. Moreover, gene ontology, biological pathways, and other molecular signature gene set databases have been integrated into CMS, enabling comparison (via methylation) of functional and biological correlated genes across different cancer types, and examining systemic alteration at biological pathway, function and interaction network levels. Users can upload their methylation profiles (generated from next-generation sequencing technologies in 100 bp resolution) or gene set to observe differential methylation by comparing with our unique collection of tumors. Also, users can download methylation intensities from a region of interest or entire genome for further analysis (by click the link in “Resources” page of the website). With CMS, biologists can access any gene of interest, examine and discover epigenetically significant phenomenon, such as (but not limited to) methylation difference between tumor types, genes with correlated methylation profiles and concordance, differentially methylated genes within a pathway, comparison of DNA methylation and histone modification marks.

## Results

CMS integrates database (from genome-wide methylation sequencing data of human cancers), web interface technology, and powerful statistical and analytical functions together, enabling genome-wide methylation profiles visualization and meaningful biological phenomenon discovery of human cancers (Figure S1 in File S1).

### Genome-wide MBDCap-sequencing of endometrial and breast cancer

A total of 232 clinical samples and cell lines derived from human breast and endometrial cancer cohorts were processed and deposited into the database. Among them, 77 are breast tumors, 10 normal breast samples, 41 breast cancer cell lines (ICBP [11]), 92 endometrial tumors (71 non-recurrent samples and 21 recurrent samples) and 12 normal endometrial samples. MBDCap-sequencing technology was used to detect the methylated regions. Methylated fragments, bound to a methyl-CpG binding domain protein, were eluted for sequencing with the Illumina/Solexa Genome Analyzer II. Approximately 12.7 billion sequence reads were generated and 52% reads were mapped to unique genome locations. Genome-wide sequencing of DNA methylation of this large set of clinical samples and cell lines made this a unique study of tumor methylome profiles (Figure S1 in File S1). Data from more than 1000 clinical samples, including ovarian, oral, colorectal, hepatocellular carcinoma, lung, and prostate cancers, will eventually be deposited into this database.

### Design of web interface and database

The web interface was developed in Java using the SideCache [12] framework, supported by a publically available JavaScript graphics library (http://www.walterzorn.de/) for graphic and image rendering. CMB website is deployed in an Apache Tomcat web server (http://tomcat.apache.org/), and supported by a MySQL database of methylation data (Figure S1 in File S1). The function and analytic methods imbedded in the framework were written in R script. In addition, a web Service API was also implemented to allow integration with other genome websites. This web interface was fully tested in Firefox, and is well compatible with Safari and Chrome. It is also compatible with IE with one disabled function (see Visualization of methylation datasets section).

### Visualization of methylation datasets

CMS can be visualized in two distinct modes: genomic view and gene centric view.

#### Genomic View

**Figure 1:**
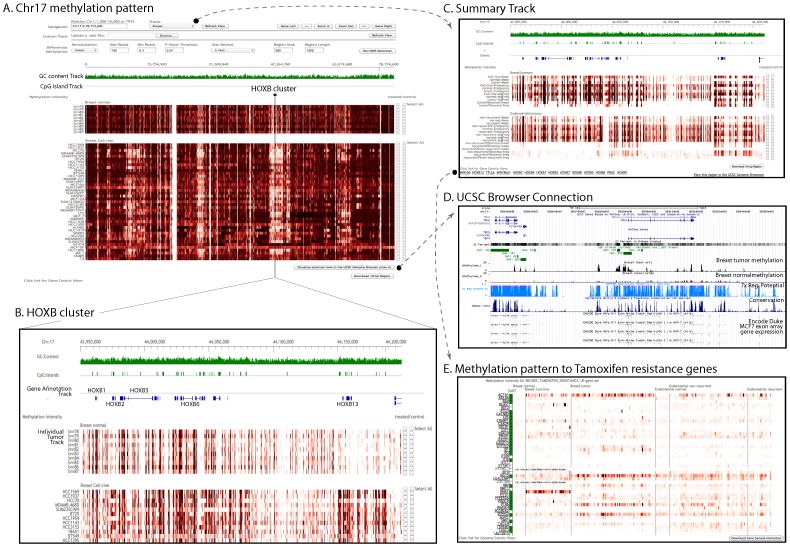
Genomic view of CMS. This webpage is designed for the genome-wide visualization and analysis of methylation intensity (A, B, C). Methylation intensity is pre-calculated for a 100 bp bin size and is shown using a red gradient heatmap. A variety of genomic annotations and functional toolbars give users more options in browsing the webpage. Statistical methods were imbedded, including DMR analysis (A) and statistical calculation (C). Links to UCSC genome browser (D) and to gene view (E) are available for further analysis.

The genomic view is for the genome-wide visualization and analysis of methylation intensity (Figure 1).

Different types of data tracks were implemented for the genomic visualization functions (Figure 1A, B): Genomic coordinate track (the genomic location of the visualized region, including chromosome, region start and end positions); GC content track (the GC percentage at the genomic position, calculated in 100 bp resolution); h3k4me1 histone modification track of GM12878 cell-line (obtained from UCSC genome build hg19, wgEncodeBroadHistoneGm12878H3k4me1StdSig.bigWig table, liftover to hg18); sequence conservation tracks from UCSC (obtained from UCSC genome build hg18); CpG island track (obtained from UCSC genome build hg18, http://genome.ucsc.edu); Gene annotation track (including gene start and end positions, gene symbol and accession number); and methylation intensity track(s) (the methylation intensity is represented by color depth, dark red corresponds to high methylation value, white means low or no methylation value, in 100 bp resolution). Detailed annotation is shown in floating-tip view when a user moves mouse over GC content, CpG island and gene annotation tracks. A single-click in the methylation profiles track(s) can generate a popup dialog with methylation intensity (reads number for that particular position, this function is disabled in IE). The methylation intensity track is flexible with several options (selected from “Tracks” drop-down button in the toolbar). Generally there are two kinds of methylation intensity tracks that users can choose to display – *individual* or *summary* tracks. An individual track shows the genome-wide methylation intensity at 100 bp bin size of each tumor/normal sample selected. Users can choose to display one tumor only (e.g., breast or endometrial), or all tumors together. Summary track (Figure 1C, see Embedded statistical methods section below) contains global statistics of mean, frequency and difference from all tumors.

A collection of well-designed functional tool-bars is included in this webpage. Users can navigate around the genome by zooming in and out, moving left or right along the genomic direction, or moving to neighboring genes. Users can search gene/region of interest by directly typing gene symbols or region coordinates.

DMR analysis (see Embedded statistical methods section below) was implemented in the genomic viewer. In DMR function, users can select candidate samples by marking the check-boxes in the methylation intensity track(s), and then fill in the necessary parameters (see Materials and Methods). Default values are preselected. The DMR will output a file in a tab-delimited text format (see Materials and Methods). All the output files will be generated on-demand and efficiently, but may be limited by the download speed of the user's network.

Links to UCSC genome browser were generated (Figure 1D, see Visualization of DNA methylation and histone modification data section for example of use). A list of genes included in the current genomic region is shown at the bottom-left of the genomic view webpage, and links are created to access the gene centric view for the particular genes (Figure 1E).

#### Gene Centric View

**Figure 2:**
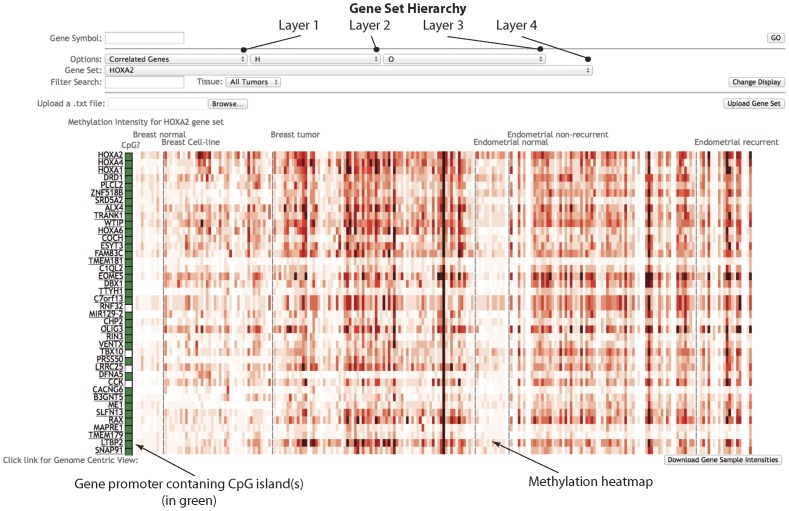
Gene centric view of CMS. This webpage is designed for visualization and analysis of methylation intensity at the gene level. In the toolbar, four layers of options are available to enable specific selections gene sets. Methylation intensities for promoter regions of genes (+/− 2 kb around TSS region) were pre-calculated and were shown using a red gradient heatmap. A white/green box on the side of gene symbol shows the promoter regions of this particular gene with or without CpG island(s). Clicking on the gene symbol on the left side of the heatmap panel will bring the user back to the genomic viewer centered on the selected gene, allowing visualization of detail methylation patterns.

An alternative way to visualize methylation data is the Gene-centric view which shows the methylation heatmap of selected gene sets (Figure 2).

In this webpage, users can type a gene symbol and visualize the methylation status of the given gene across all tumor samples, along with the top 40 most correlated genes with similar methylation patterns calculated by Pearson correlation (see Materials and Methods). Alternatively, four layers of options are available to enable selections of specific biological function, interaction network, and correlated gene sets (Figure 2). There are eight primary classes of gene sets (some of them may include subsets). These are predefined in the first layer, including Correlated genes (see Materials and Methods), Chromosomal, Gene Ontology, Perturbation sets, Biological Pathways, microRNAs, Transcription Factors, and Cancer gene neighborhood. The primary gene set names and their sources are listed in Table 1 [13]–[19]. Methylation status of a chosen gene set can be visualized for all tumors within CMS, or any tumor types of user's selection. Large gene sets may slow down the methylation heatmap rendering time, thus it is preferable to choose smaller gene sets to start the process. The "Filter Search" option allows a user to find all gene sets (except those among the “Correlated Genes”), which contain the words in the search field.

**Table 1:** Eight classes of gene set names and their sources.

Gene Set Name	Description	Source	Ref
Chromosomal	Genes with a given chromosomal location	MSigDB	[13]
Gene Ontology	Gene sets derived from gene ontology terms in all three GO categories	MSigDB	[13]
Perturbation sets	Gene sets obtained from chemical and genetic perturbation	MSigDB	[13]
Biological Pathways	Genes derived from various pathway systems	MSigDB,WikiPathways, Reactome, KEGG, NCI Nature, BioCarta and HumanCyc	[13]–[19]
microRNAs	Genes that regulated by miRNAs	MSigDB	[13]
Transcription Factors	Genes that regulated by transcription factors	MSigDB	[13], Version 7.4, http://www.gene-regulation.com/
Cancer gene neighborhood	Genes that associated with 380 cancer genes.	MSigDB	[13]
Correlated genes	Genes that are correlated based on methylation status of the CMB 191 tumors		Pearson Correlation, top 40 or >0.4.

In the heatmap panel, the methylation intensities were pre-calculated by averaging the normalized (linear normalization, see Materials and Methods) reads number within +/− 2-kb of a transcription start site (TSS) and were stored in the MySQL database. Different from the genomic view, the gene centric viewer is organized as follows: tumor or normal samples are placed in columns, and genes are rows, similar to common microarray format. The heatmap color scale of gene centric view is the same as that of genomic view. Promoter regions with or without CpG island(s) are annotated with a white/green box on the side of gene symbol. The heatmap panel makes it possible to visualize different/similar/special methylation profiles (See Discovery by use of CMS section) between different tumor types, or among the genes within similar biologically significant categories.

Clicking on the gene symbol on the left side of the heatmap panel will bring the user back to the genomic viewer centered on the selected gene, allowing visualization of detail methylation patterns in the promoter, exon, intron and its neighboring regions.

### Input and output

In genomic view, users who wish to visualize and analyze their own data can enable a custom track. The data submitted by users are private, session-based (not stored after the end of session), and not viewable by others. One the other hand, for a region of interest (less than 1 Mbp, shown in the bottom-right of the web page of genomic view), users can download the reads information (in BED format) for further analysis.

In gene centric view, we also provided a file upload option to allow users to upload their customized gene sets (official gene symbols only). The custom gene set will be shown as “User Input” in the drop-down button of the Gene Set layer. Users can also download the methylation intensity of the current heatmap panel by clicking the button in the bottom-right of the webpage.

### Embedded statistical methods

Hypermethylation of the CpG islands of the gene promoter is one of the most frequent alterations leading to cancer, and an important epigenetic mechanism for gene silencing. To enable the detection of the differential methylation regions between two sample groups, the DMR identification function was embedded in the framework. In CMS, individual methylome tracks (including user uploaded custom-track) or summary tracks can be assigned to one of two groups, defined as “treated” and “control” (see Visualization of methylation datasets section). A DMR detection algorithm, based on *t*-test, Wilcoxon test or Pearson correlation can be selected to assess the significance of differential methylation up to 1 mega base-pairs. The description of the DMR algorithm is provided in the Materials and Methods.

In addition to the DMR function, we also designed summary tracks to visualize the averaged methylation intensities and to reveal intrinsic characteristics of each tumor group. Three types of summary tracks are displayed together as shown in Figure 1C, they are: (a) Mean track, which provides average methylation status over a particular group of samples. Currently the summary statistics are evaluated over i) all samples, ii) normals, tumors and cell lines of breast, and iii) endometrial non-recurrent tumors, recurrent tumors, and normal of endometrial; (b) Methylation frequency track (see Materials and Methods). Mean and frequency tracks provide insight to whether the methylation change is from majority samples or minority samples with large methylation intensity; (c) Difference track, which visualizes differential methylation by mean/frequency difference between groups of samples at each bin size, such as tumor vs normal-mean for breast, and non-recurrent/recurrent vs normal-freq for endometrial.

### Tumor specific methylation profiles

The tumorigenesis mechanisms are different among cancers, therefore it is important to find genetic/epigenetic differences for further analysis. Here we used the HOXB2 (human homeobox B2) gene, a member of the Antphomeobox family that encodes a nuclear protein with a homeobox DNA-binding domain, and a known gene associated with tumor growth and invasiveness [20], [21] as an example to illustrate how CMS is able to determine tumor specific methylation profiles for breast and endometrial cancers.

**Figure 3:**
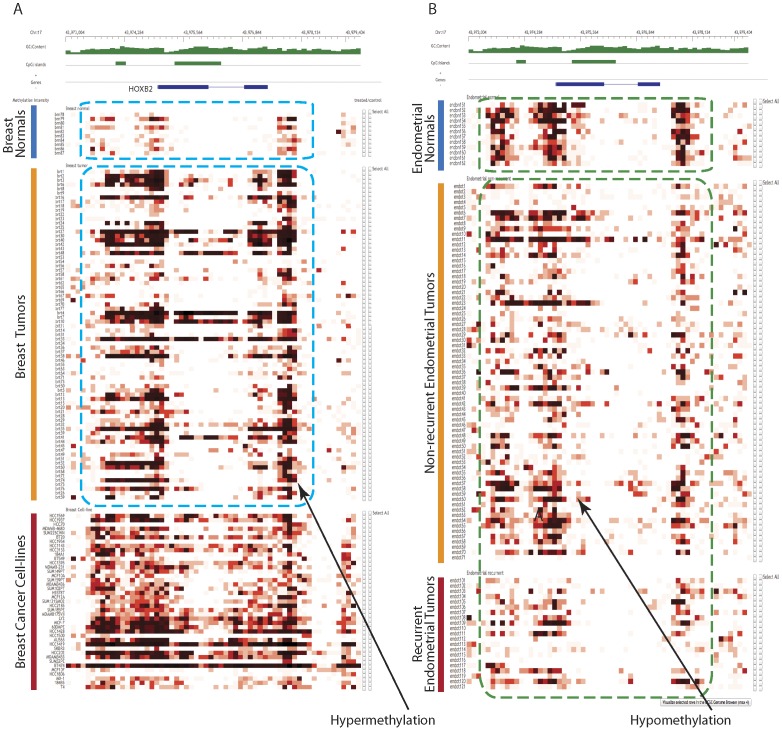
Discovery of tumor specific methylation profiles. HOXB2 was hypermethylated in breast tumors compared with breast normal (A), while hypomethylated in endometrial cancer tumors compared with endometrial normal (B).

In genomic view, users can type HOXB2 in the navigation box, and choose “All Tumors” in the Tracks drop-down box, then click the “Refresh View” button. For a better view of the methylation profiles, users can click the “zoom in” button four times. Clearly hypermethylation was found between breast tumors and normal (Figure 3A), including four regions (*p*-value<0.01) calculated by the DMR function using default parameters. However, hypomethylation was found between endometrial tumors and normal tissues (Figure 3B), including one region with *p*-value<10^−4^ (Table S1 in File S1). Additionally, users can browse the summary track by selecting “All summaries” in the tracks drop-down box. The mean track, representing hundreds of individual tracks, simplifies the visualization of differentially methylated regions giving a more intuitive result. Besides genes that are hypermethylated only in breast tumors (compare with breast normals), users can also find genes that are hypermethylated only in endometrial tumors (compare with endometrial normals) (such as CCDC81, Figure S2 in File S1), and in both tumors (such as SOX11, Figure S3 in File S1).

### Similar methylation profiles among biologically related genes

**Figure 4:**
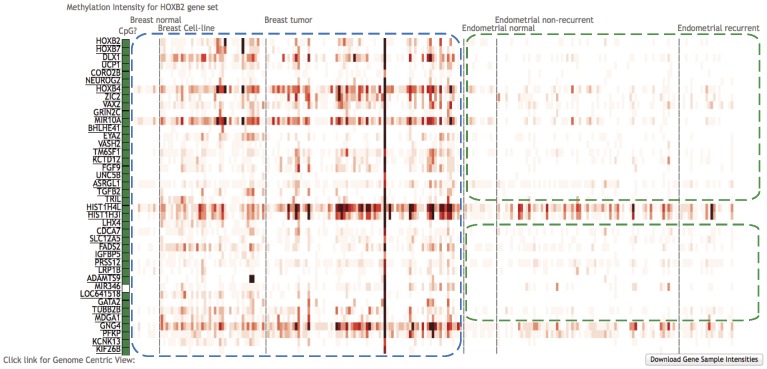
Discovery of methylation correlated genes. Gene set with similar methylation profiles of HOXB2 were found by choosing the “Correlated gene” gene sets in the gene centric view. Most of the genes are hypermethylated in breast tumors (blue dash box), and with no significant difference in endometrial samples (green dash box).

Homeodomain genes encode transcription factors that affect differentiation and proliferation during development. In the human genome, four clusters of homeodomain genes (HOXA, HOXB, HOXC and HOXD) are distributed on chromosomes 7p15, 17q21, 12q13 and 2q31, respectively. Non-clustered homeodomain genes are distributed throughout the genome. One direct question is “what are the other genes that display the same methylation pattern as that of HOXB2, perhaps sharing the same methylation mechanism?” Continuing with the previous process at the genomic view, users can click the gene link in the left-bottom to get the gene centric view. The top 40 correlated genes of HOXB2 are shown in Figure 4. Most of them have a similar methylation profile as HOXB2, which is hypermethylated in breast tumors (Figure 4, blue dash box), and is either hypomethylated or shows no difference in endometrial tumors compare to normal tissues (Figure 4, green dash box).

In the 40 correlated genes, three of them belong to HOXB gene family (HOXB2, HOXB4 and HOXB7), three genes contain homeodomain (DLX1, LHX4, and VAX2) and two of them belong to HIST gene family (HIST1H3I and HIST1H4L). A similar methylation profile of the genes within the same gene family defines the methylation concordance, which may lead to synchronized gene silencing. Moreover, users can also find the genomic neighbors of HOXB2 by choosing “Chromosomal” gene set in layer one, “chr17” in layer two, “q” arm in layer three and “chr17q21” in layer four. This cytoband covers 287 genes, and harbors the HOXB gene cluster including three genes (HOXB2, HOXB4 and HOXB7) overlapped with the 40 HOXB2 correlated genes. Notice that the three genes are both in the same gene family and the same genomic location, which may indicate significant biological concordance for those genes. Users may find missing values for several genes in “Chromosomal” gene sets, due to the lack of transcript annotation within NCBI Reference Sequence (RefSeq) release contained in UCSC genome browser or obsolete gene symbols. This phenomenon also happens for the other 7 classes of categories.

### Differentially methylated gene sets within a pathway

**Figure 5:**
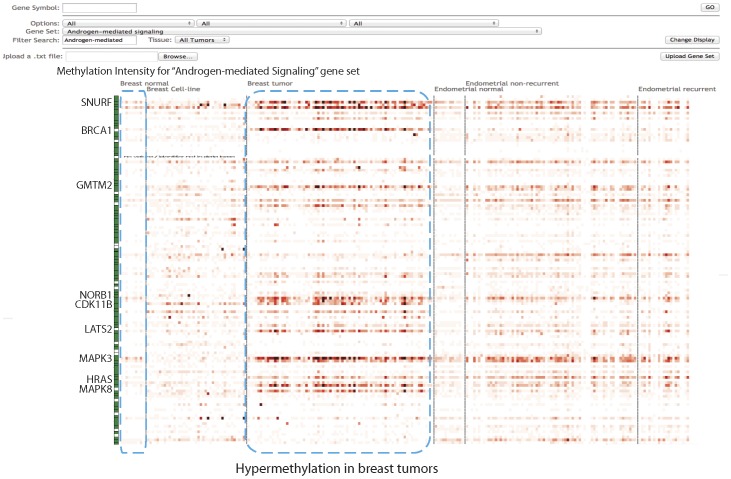
Discovery of differentially methylated gene sets within a pathway. The “Androgen-mediated Signaling” gene set which contains HOX cluster genes were selected as an example. Several genes within the blue dash box are hypermethylated in breast tumors compared to normal tissues, while others show no significant difference. For endometrial samples, no significant difference is found for any of the gene between tumors and normals.

To examine the systemic change of biological pathway activity or functions that related to HOXB2 or other HOX family genes, we examined the following gene sets to illustrate the functional discovery by using CMS tools. HOXB13, a HOX family member resides in the cluster of HOXB2 and shows a similar methylation pattern as HOXB2. HOXB13, is also a member of “androgen-mediated pathway”, as shown in Figure 5. It shows a distinct hypermethylation pattern among breast tumors, but not breast cancer cell-lines and endometrial cancers. Specifically, the distinct hypermethylation pattern of BRCA1, SNURF, GMTM2, NROB1, CDK11B, LATS2, HRAS, MAPK3, RPS6KA3 and EGR1 demarcate the cluster's methylation status of breast tumor (not cell-lines), along with HOXB13.

We also compared methylation profiles for Tamoxifen resistant genes [22], and identified several hypermethylation genes in breast tumors, such as ACTA1, ISG15, PTK6 and SEPHS2 (Figure 1E). Most of them didn't show significant difference in endometrial samples.

### Visualization of DNA methylation together with histone modification data

A convenient URL link to UCSC opens the current genomic region in the UCSC genome browser for users who wish to view other genomic data (bottom-right of the genomic view, Figure 1D). Alternatively, users can select up to four intensity tracks and view those tracks together with other default tracks in the UCSC genome browser.

**Figure 6:**
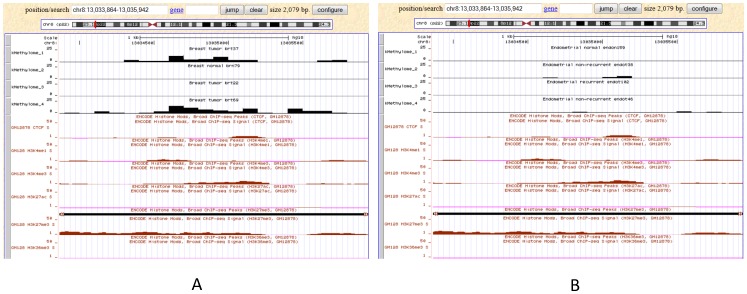
Visualization of DNA methylation and histone modification data. The TSS region of DLC1 is used as an example. 4 samples were randomly selected by marking the check box on the right side of the webpage for breast samples (e.g., brn80, brt22, brt69 and brt37). The “full” option for every custom track and the Broad Histone tracks was selected for the comparison of DNA methylation and histone modification marks. Similar results were obtained as previous report [4]. An exception (the 3^rd^ track, brt22) was found which shows patient specific patterns (A); and there was no increased methylation found for endometrial samples (B).

For example, DLC1 gene was reported to have increased DNA methylation at its transcription start site (TSS) region, while decreased histone modification in H3K4me1, H3K4me3 and H3K27ac at TSS region [4]. Users can type DLC1 in genomic view webpage, and visualized the TSS region (Chr8:13,033,864-13,035,942) by clicking the “zoom in” and “move” buttons. We can get the overall impression that breast tumors are hypermethylated relative to breast normals, while endometrial tumors show no difference relative to endometrial normals. Users can pick up 4 samples randomly by marking the check box on the right side of the webpage for breast samples (e.g., brn80, brt22, brt69 and brt37), and then click the “Visualize selected rows in the UCSC Genome Browser button” in the bottom-right of the webpage, to open a UCSC webpage. To compare with the histone modifications tracks, users need to select “full” for every custom track and the Broad Histone tracks. The histone modification tracks (Figure 6) are in accordance with previous report [4] although those data may not come from breast cancer. Custom tracks (DNA methylation) of breast cancers have increased methylation (similar to previous finding) with an exception (the 3 rd track, brt22), which shows patient specific patterns (Figure 6A). Not surprisingly, there was no increased methylation found for endometrial samples (Figure 6B).

## Discussion

In our studies, HOXB2 was used as an example to find out biologically significant information by use of CMS. This is because HOXB2 was found as a regulator of tumor growth in breast cancer [23]. Interestingly, we found HOXB2 was hypermethylated in endometrial normal tissues compared with endometrial tumors (Figure 3B). In previous study, HOXB2 was reported to be important in endometrial normal cells [24]. Moreover, HOXB2, HOXB4 and HOXB7 together showed the key function in lung cancers [25]. In our study, we also identified that those 3 genes are correlated in their methylation profiles. This might suggest that these three genes function together in breast and endometrial cancers. Furthermore, HOXB13 and BRCA1 are all from “androgen-mediated pathway” (Figure 5), and are all found to be hypermethylated in breast tumors than normal tissues in our study. This is also consistent with previous report that HOXB13 acts as repressor of androgen receptor signaling in prostate cancer, which may affect BRCA1 (cofactor associated with AR) [26].

There have been several epigenetics websites available in previous published reports. One of the most famous is Roadmap Epigenomics Project (REP) (http://www.roadmapepigenomics.org/). This project was composed of a group of various databases, browser/visualization tools, and bioinformatics tools. Users can either view many kinds of epigenetic marks in their browser (e.g. UCSC REP, http://www.epigenomebrowser.org/), or download the data from one of the data repositories (http://www.ncbi.nlm.nih.gov/epigenomics). Compared with CMS, REP is more comprehensive in both data variety and derivative tools. However, CMS is designed to provide clinical tumor samples, and we have additional statistical methods specifically for genome-wide analysis and comparison of those samples (like DMR detection and correlated genes function).

## Conclusion

In this study, we proposed CMS for visualization and analysis of methylation datasets for cancers. A large number of datasets were collected and processed into our database. Several statistical tools were imbedded for data analysis. Visualization was developed through a Java based web interface. Useful discoveries were made by the extensive application of this framework. A large dataset, a variety of tools and extensive application with biological and translational significance makes this framework powerful and unique in cancer methylation research.

## Materials and Methods

### Tissue Specimens, cell line and MBDCap-seq

Tissue specimens were obtained as part of our ongoing work on characterizing molecular alterations in endometrial and breast carcinomas.

The ICBP breast cancer cell lines genomic DNA was isolated by the QIAamp DNA Mini Kit (Qiagen) following the manufacture's protocol. Genomic DNA of breast cell lines was procured through the Integrative Cancer Biology Program (ICBP) of the National Cancer Institute.

MBDCap libraries for sequencing were prepared following standard protocols from Illumina (San Diego, CA). MBDCap-seq libraries were sequenced using the Illumina Genome Analyzer II (GA II) as per manufacturer's instructions. Sequencing was performed up to 36 cycles for mapping to the human genome reference sequence. Image analysis and base calling were performed with the standard Illumina pipeline.

### Data preprocessing

Sequencing reads were mapped by the ELAND algorithm (Illumina Inc, San Diego, CA). Reads were in 36 base pair lengths, and uniquely mapped reads were mapped to the human reference genome (hg18), with up to two mismatches. Genome-wide methylation status at 100 base-pair resolution was evaluated. In each 100-bp bin, the methylation intensity was quantified by accumulating the read numbers in which whole or part of the read was located within the bin. The 100 bp resolution sequence read counts were deposited to a MySQL database table for visualization and analysis at the genomic level, such as the DMR function.

### Differentially Methylated Regions (DMRs) algorithm, parameters, and output file format

Two kinds of normalization methods will be used when DMR function is called in the genomic view.

#### Normalization method

The methylation intensity was normalized based on the unique read numbers for each sample by either the linear method or quantile method. The following equation was used for linear normalization:
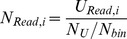
(1)Where *N_Read,i_* is the normalized read number of the *i* ^th^ bin, and *U_Read,i_* is the unique mapped read number of the *i* ^th^ bin, *N_U_* is the total unique mapped reads number. *N_bin_* is the total bin number of human.

In quantile normalization, the distribution of methylation intensity of the first group is used as the reference, and the methylation level of the second group is transformed. The transformation can be formulated as follows:
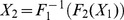
(2)Where *F*_1_ is the distribution of the first group and *F*_2_ is the distribution of the second group.

#### DMR detection method

Suppose we have two groups *A* and *B*, and the sample number is *S_A_* for group *A*, and *S_B_* for group *B*. For a given region *R* (which includes *m* bins, and start at the *s* ^th^ bin), the average methylation level is
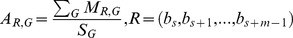
(3)for group *G* 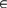 {*A*, *B*}. In Equation 3, *A_R,G_* is the average methylation level of group *G* at region *R*, *M_R,G_* is the methylation levels of each sample of group *G* at region *R*. We then used statistical methods (see below) to compare if the methylation level of the region is significantly different between those two groups
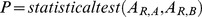
(4)


For each DMR, we defined hyper-methylation as the average methylation enrichment if the region of group *A* is higher than group *B*, and vice versa (hypo-methylation). Three statistical test methods were used: Paired *t*-test, Wilcoxon test, and Pearson correlation coefficient.

### DMR algorithm parameters

1. Normalization methods: two normalization methods were included: linear normalization and quantile normalization. Default method is linear normalization.
2. Max Reads: the maximum threshold for methylation intensity (for 100 bp bin size). The methylation levels larger than the threshold will be removed. Default value is 100.
3. Min Reads: the minimum threshold for methylation intensity (for 100 bp bin size). The methylation levels smaller than the threshold will not be considered for DMR calculation. Default value is 0.3.
4. *P*-value Threshold (significance level): the *p*-value required for DMR detection for the statistical methods mentioned below. We suggested *p*-value less than 0.05 for *t*-test or Wilcoxon test, and less than 0.3 (low correlation coefficient correspond to high difference) for Pearson correlation coefficient. Default *p*-value is 0.01.
5. Stat Method: the statistical method used for the DMR detection. Three options were included: *t*-test, Wilcoxon test and Pearson correlation coefficient. Default method is *t*-test.
6. Region Step: the moving window (region) step. Default step is 500 bp.
7. Region Length: the window size of the specific region that is used for the comparison between two groups of samples, this window size shall be larger than bin size to allow large enough data points to be tested. The entire genome is scanned by this window size, with a moving step defined above. Default length is 1000.

### DMR output file format

1. Chromosome, region start and region end (1–3 columns): the genomic coordinates of the DMR region.
2. Type (4 th column): DMR type:
  - Hypermethylation (treated samples have higher methylation than control samples).
  - Hypomethylation (treated samples have less methylation than control samples).
3. *P*-value (5 th column): Calculated *p*-values from the statistical test. Only the DMRs with *p*-value smaller than the *P*-value Threshold defined above will be outputted.
4. Methylation difference (6 th column): the difference of averaged methylation intensity between treated and control samples. Positive value corresponds to DMR Type 1, and negative value represents DMR Type 2.
5. Methylation ratio (7 th column): the percentage of methylation difference between treated and control samples, calculated by the methylation difference divided by averaged methylation intensity of control samples.

### Frequency track of Methylation Intensity

Two algorithms are provided here for methylation frequency calculation:

1. Simple Methylation Frequency: For each bin (bin size of 100 bp), the methylation frequency is the occurrence frequency of methylation intensity greater than 2 for the same bin along all samples with a group of interest. Because most of the methylation intensity is less than 2 (bin size of 100 bp at CMB database), the high methylation frequency could be considered an important methylated position.
2. Segmented Methylation Frequency (segFreq): The aim of the segmented methylation frequency is to reduce the noise due to some erroneous read count of certain bins (100 bp). Similar to Simple Methylation Frequency calculation, except a segmentation algorithm is applied before counting occurrence of methylation greater than 1.0. The segmentation algorithm is provided briefly here: i) all methylation data are thresholded at read count of 1, and converted into binary runs; ii) find all runs of 1 s; iii) if adjacent runs of 1 s are no farther than 200 bp away (1 bin apart), connect them (remove single bin of count 0 within a long run of 1 s); and iv) if run-length of 1 s is 1 (single bin) and it is bin count is less than 3, remove the bin. The simple methylation frequency calculation will be performed then.

### Calculation of correlated genes of gene sets

In the heatmap of gene centric view, each row stands for methylation pattern of a particular gene. The pattern is consisted of a group of averaged methylation value around +/−2 kb of TSS region of this particular gene across different tumor samples. We provide up to 40 of the most correlated genes (Pearson correlation, at least *ρ*≥0.4. Correlation of 0.4 is chosen because the probability of *p*>0.4 for two normally distributed random variables with *N* = 232 is less than 10^−10^).

Other gene sets were selected from various sources (see Table 1). Methylation statuses of genes with each set can be displayed. No statistical assessment is performed, other than visualization, for association of biological functions of gene sets to methylation patterns.

## Supporting Information

Figure S1 **The database (from genome-wide methylation sequencing data of human cancers), web interface technology and embedded powerful statistical and analytical functions were integrated as a framework for the visualization and analysis of methylation profiles of human cancers.**
(PDF)

Figure S2 **Extension of CMS applications: Discovery of tumor specific methylation profiles.** CCDC81 has no significant difference between breast tumors and breast normal tissues, while it is hyper-methylated in endometrial tumors compared with endometrial normal tissues.
(PDF)

Figure S3 **Extension of CMS applications: Discovery of tumor specific methylation profiles.** SOX11 was hypermethylated in breast tumors compared with breast normal tissues, and was also hyper-methylated in endometrial tumors compared with endometrial normal tissues.
(PDF)

Table S1 **DMR regions of Breast and Endometrial cancers for HOXB2 gene.**
(PDF)
