# Supplementary figures and images for: CMS: A Web-Based System for Visualization and Analysis of Genome-Wide Methylation Data of Human Cancers
Source: PLoS One. 2013 Apr 22;8(4):e60980. doi: 10.1371/journal.pone.0060980 (PMC3632540; doi:10.1371/journal.pone.0060980)

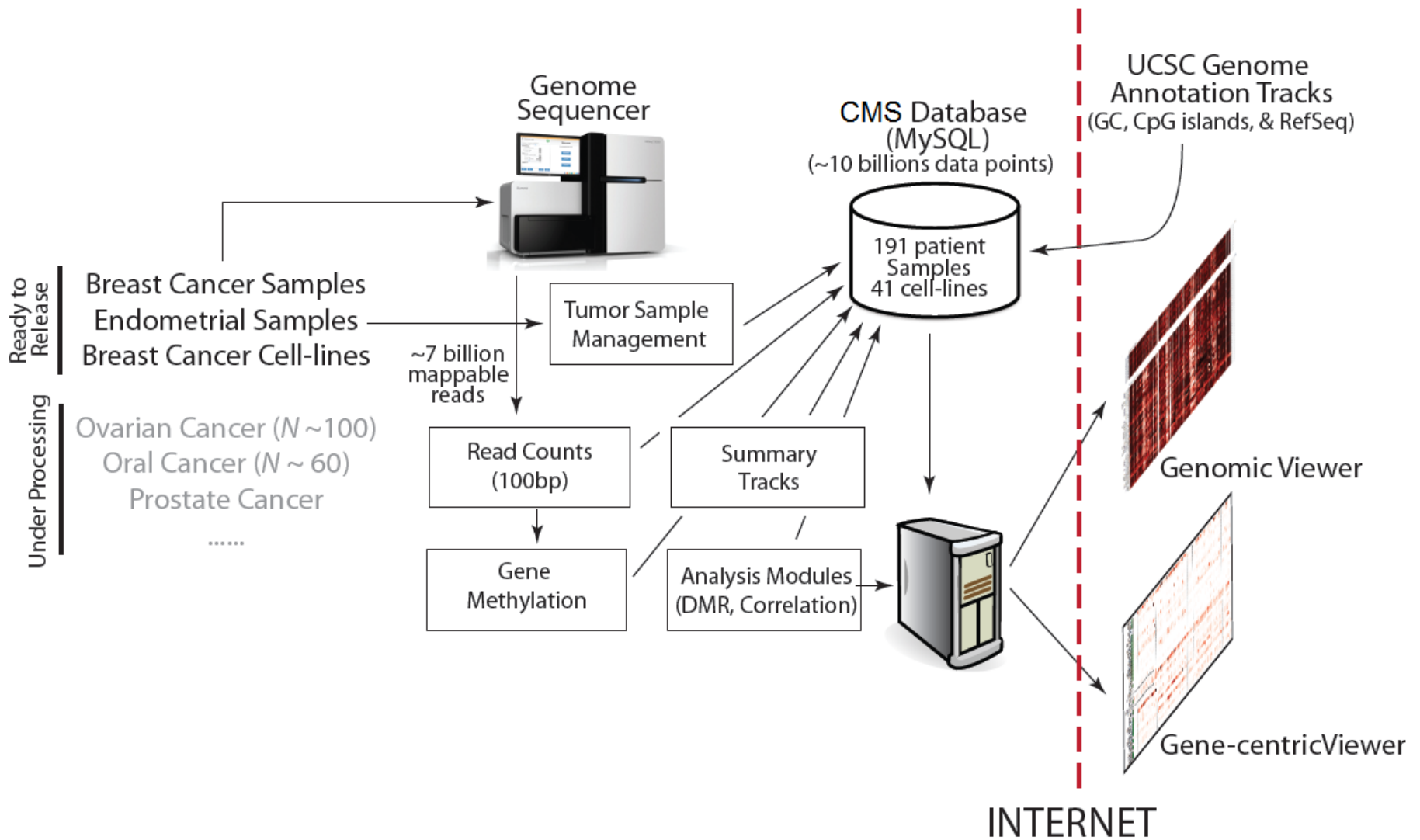

Supplementary figure S1

Supplement: Figure S1 — The database (from genome-wide methylation sequencing data of human cancers), web interface technology and embedded powerful statistical and analytical functions were integrated as a framework for the visualization and analysis of methylation profiles of human cancers. (PDF) [file pone.0060980.s001.pdf]

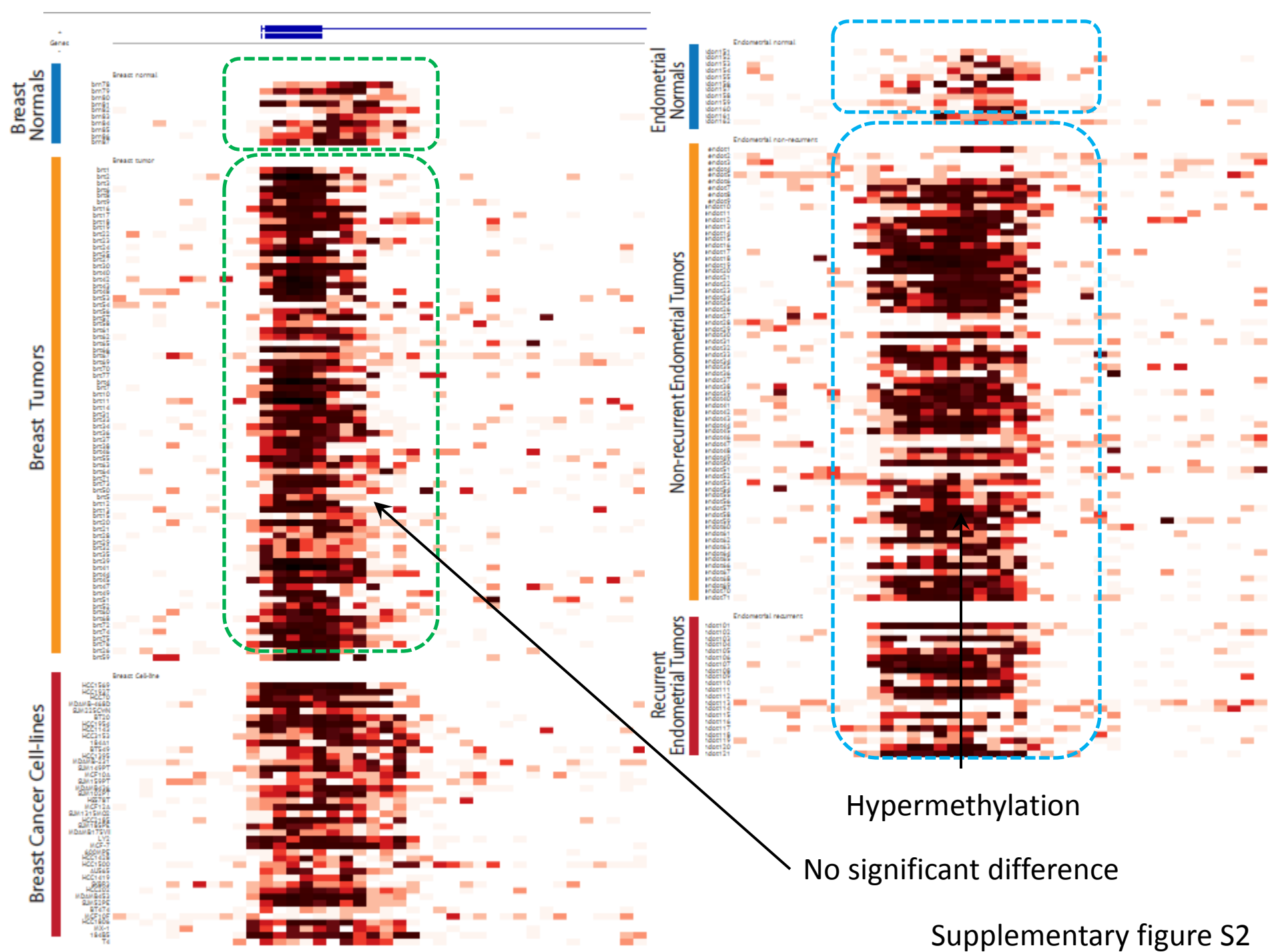

Supplementary figure S2

Supplement: Figure S2 — Extension of CMS applications: Discovery of tumor specific methylation profiles. CCDC81 has no significant difference between breast tumors and breast normal tissues, while it is hyper-methylated in endometrial tumors compared with endometrial normal tissues. (PDF) [file pone.0060980.s002.pdf]

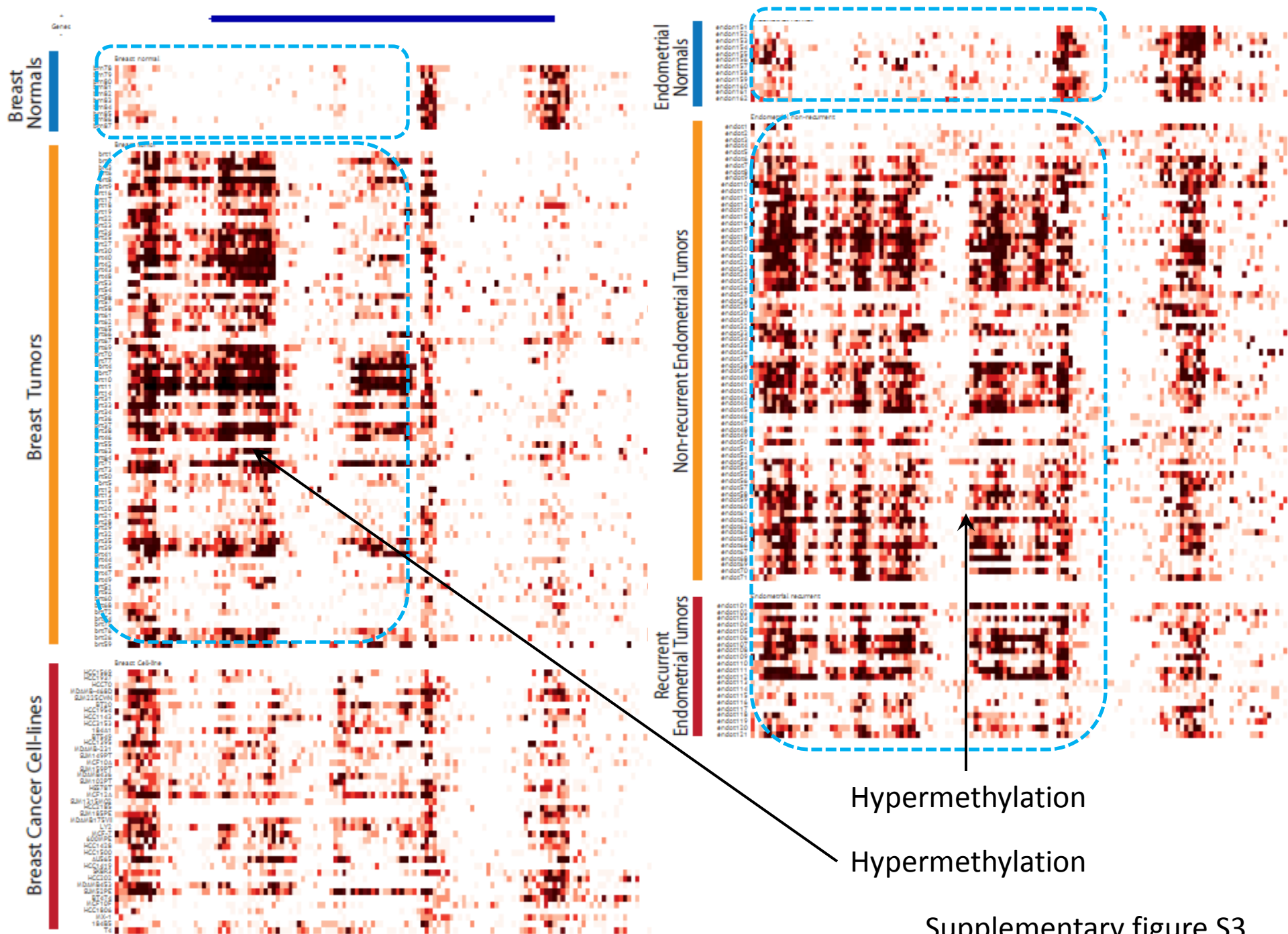

Supplement: Figure S3 — Extension of CMS applications: Discovery of tumor specific methylation profiles. SOX11 was hypermethylated in breast tumors compared with breast normal tissues, and was also hyper-methylated in endometrial tumors compared with endometrial normal tissues. (PDF) [file pone.0060980.s003.pdf]
