# Supplementary material for: CMS: A Web-Based System for Visualization and Analysis of Genome-Wide Methylation Data of Human Cancers
Source: PLoS One. 2013 Apr 22;8(4):e60980. doi: 10.1371/journal.pone.0060980 (PMC3632540; doi:10.1371/journal.pone.0060980)
Supplement: Table S1 — DMR regions of Breast and Endometrial cancers for HOXB2 gene. (PDF) [file pone.0060980.s004.pdf]

Supplementary table S1. DMR regions of Breast and Endometrial cancers for HOXB2 gene.

	Chromosome	Region start	Region end	Type	P-value
Breast DMR 1	chr17	43,973,504	43,974,504	1	0.0034
Breast DMR 2	chr17	43,974,504	43,975,504	1	0.0024
Breast DMR 3	chr17	43,975,504	43,976,504	1	$1.00 x 10^{-4}$
Breast DMR 4	chr17	43,976,504	43,977,504	1	0.0051
Endometrial DMR 1	chr17	43,974,504	43,975,504	2	$7.00 x 10^{-4}$
